# Italian Recommendations for Placental Transfusion Strategies

**DOI:** 10.3389/fped.2018.00372

**Published:** 2018-12-03

**Authors:** Stefano Ghirardello, Mariarosaria Di Tommaso, Stefano Fiocchi, Anna Locatelli, Barbara Perrone, Simone Pratesi, Paola Saracco

**Affiliations:** ^1^Neonatal Intensive Care Unit, Department of Clinical Sciences and Community Health, Fondazione IRCCS Cà Granda Ospedale Maggiore Policlinico, Milan, Italy; ^2^Health Sciences Department, University of Firenze, Careggi University Hospital, Florence, Italy; ^3^Neonatology and Neonatal Intensive Care Unit, ASST Grande Ospedale Metropolitano Niguarda, Milan, Italy; ^4^Obstetrics and Gynecology Unit, School of Medicine and Surgery, University of Milano Bicocca, Milan, Italy; ^5^Division of Neonatology and NICU, Salesi Children's Hospital, Ancona, Italy; ^6^Neonatology Unit, Careggi University Hospital, Florence, Italy; ^7^Department of Pediatric Sciences, Azienda Ospedaliero-Universitaria Città della Salute e della Scienza di Torino, Turin, Italy

**Keywords:** delayed cord clamping, preterm newborn, umbilical cord milking, neonatal resuscitation, recommendation, cord blood banking, twin, HIV pregnancy

## Abstract

At delivery, if the cord is not clamped, blood continues to pass from the placenta to the newborn during the first minutes of life, allowing the transfer of 25–35 ml/kg of placental blood to the newborn, depending on gestational age, the timing of cord clamping, the position of the infant at birth, the onset of respiration, and administration of uterotonics to the mother. However, deriving benefits from delayed cord clamping (DCC) are not merely related to placental-to-fetal blood transfusion; establishing spontaneous ventilation before cutting the cord improves venous return to the right heart and pulmonary blood flow, protecting the newborn from the transient low cardiac output, and systemic arterial pressure fluctuations. Recent meta-analyses showed that delayed cord clamping reduces mortality and red blood cell transfusions in preterm newborns and increases iron stores in term newborns. Various authors suggested umbilical cord milking (UCM) as a safe alternative when delayed cord clamping is not feasible. Many scientific societies recommend waiting 30–60 s before clamping the cord for both term and preterm newborns not requiring resuscitation. To improve the uptake of placental transfusion strategies, in 2016 an Italian Task Force for the Management of Umbilical Cord Clamping drafted national recommendations for the management of cord clamping in term and preterm deliveries. The task force performed a detailed review of the literature using the GRADE methodological approach. The document analyzed all clinical scenarios that operators could deal with in the delivery room, including cord blood gas analysis during delayed cord clamping and time to cord clamping in the case of umbilical cord blood banking. The panel intended to promote a more physiological and individualized approach to cord clamping, specifically for the most preterm newborn. A feasible option to implement delayed cord clamping in very preterm deliveries is to move the neonatologist to the mother's bedside to assess the newborn's clinical condition at birth. This option could safely guarantee the first steps of stabilization before clamping the cord and allow DCC in the first 30 s of life, without delaying resuscitation. Contra-indications to placental transfusion strategies are clinical situations that may endanger mother ‘s health and those that may delay immediate newborn's resuscitation when required.

## Introduction

Until 1960, delayed umbilical cord clamping (DCC) to promote placental transfusion was common; afterward, cord clamping immediately after birth became standard practice to reduce the risk of postpartum hemorrhage, although without evidence (1, 2). In the last decade, great interest has been renewed on DCC and umbilical cord milking (UCM), an active maneuver by which the content of the umbilical cord is gently squeezed toward the newborn (3).

Both techniques, referred in the text as placental transfusion strategies, allow transferring a similar amount of fetal blood, between 25 and 35 ml/kg, from the placenta to the newborn, increasing neonatal volemia, hemoglobin concentration, and blood pressure in the first days of life (4–6).

The amount of blood transferred to the newborn during DCC depends on various factors: the time of cord clamping, the mode of delivery, the position of the newborn, the beginning of spontaneous breathing and respiration, and uterine contractions (7). The ideal timing of cord clamping is likely different for every newborn, depending on how rapidly pulmonary vascular resistances reduce and placental blood fills the pulmonary vascular bed. Indeed, various researchers underlined how cord clamping should be individualized for each newborn (8–10).

Milking the umbilical cord is considered a valid alternative to DCC, as it takes 10–20 s to be performed, allowing rapid neonatal resuscitation when required. It consists of squeezing gently 20–30 cm of umbilical cord three to five times from the placenta to the newborn at a velocity of 10 cm/s (3, 5, 11). This maneuver could be carried out when the cord is intact (connected to the placenta) or after it is cut and separated from the placenta (c-UCM) (12). The combined intervention of DCC followed by UCM (13) showed a positive cumulative effect on neonatal hematological parameters in the first weeks of life, compared to DCC or UCM only, without side effects.

### Placental Transfusion Strategies and Neonatal Outcomes

In the last years, research studies and meta-analysis highlighted the favorable impact of placental transfusion strategies on short-term neonatal outcomes. Both have been associated with a lower incidence of iron deficiency at 3–6 months in term newborns (14–16). In preterm newborns DCC and UCM were associated to higher arterial blood pressure and hemoglobin at admission, reduced need for red blood cell transfusions, reduces incidence of intraventricular hemorrhage (IVH), late-onset sepsis, necrotizing enterocolitis (NEC), and mortality (17–23). Umbilical cord milking in newborns with gestational age <33 weeks reduced the risk of bronchopulmonary dysplasia and intraventricular hemorrhage (11). The most recent results deriving from a multicenter trial (APTS) (24) that enrolled to delayed or immediate clamping more than 1,500 preterm newborns with gestational age <30 weeks failed to demonstrate differences in death or major morbidity (severe brain injury, severe retinopathy of prematurity, necrotizing enterocolitis, or late-onset sepsis) between DCC and early clamping. The same working group, however, in the subsequent meta-analysis of 18 controlled trials (25) concluded for a strong and significant association between DCC and reduced hospital mortality, both in preterm and extreme preterm infants and confirmed a significant reduction in red blood cell transfusions.

The reported long-term effects of placental transfusions are still scarce; a recent neuro-cognitive follow-up study of term newborns associated DCC to improved fine motor and social domains scores at 4 years of age, particularly in males (26). Umbilical cord milking in preterm newborns was associated to higher language and cognitive scores at 2 years of corrected age compared to DCC (27).

### Placental Transfusion Strategies and Neonatal Resuscitation

Recent studies on animal models (28, 29) showed that DCC promotes a smoother cardiopulmonary transition at birth. Indeed, if the cord is clamped immediately, left ventricle suddenly loses the source of its filling, and the left ventricle preload becomes fully dependent on pulmonary venous return from the lungs. Pulmonary blood flow increases slowly after birth as the newborn starts breathing. This period between cord clamping and the onset of breathing is called “non-respiring interval,” during which hemodynamic fluctuations associated with reduced cerebral oxygenation have been demonstrated in animals (29). Otherwise, if the cord is maintained unclamped, blood coming from the placenta through the umbilical vein continues to fill left ventricle, while the onset of newborn's ventilation increases pulmonary blood flow and venous return (29). Thus, delaying cord clamping until after the start of breathing would maintain left ventricle pre-load unchanged, as demonstrated by the absence of hemodynamic fluctuations in animal models (28).

Scientific societies and neonatal resuscitation guidelines (30–34) suggest delaying the clamping of the cord by 30–60 s in vigorous preterm infants and by 3–5 min in term newborns not requiring resuscitation but recommend immediate cord clamping for apneic or limp newborns at birth (31, 32).

Therefore, “newborns requiring resuscitation” have been excluded by most research protocols and a significant percentage of infants included in DCC trials were clamped earlier than scheduled, mostly due to clinical concerns about delaying ventilation in non-vigorous infants (19, 24, 35–38). The observation by Nevill et al. (36) that preterm newborns not breathing during DCC were more likely to be intubated, have chronic lung disease, and severe intraventricular hemorrhage compared to breathing infants, underlines the importance of promoting spontaneous breathing during DCC.

When stimulation of the newborn occurs before clamping the cord, as suggested by WHO guidelines (30), most term and preterm infants start breathing spontaneously within the first minute of life (39–41).

A feasible option to promote placental transfusion in the most compromised newborns is to provide neonatal resuscitation during DCC. Recently Katheria et al. (41) randomized newborns with gestational age below 32 weeks to receive NCPAP or positive pressure ventilation during DCC ≥ 60 s or to receive DCC only; this latter group, if apneic, was dried and stimulated until the onset of spontaneous breathing. The authors demonstrated that there was no significant difference in the time from birth to the first spontaneous respiratory act between the two cohorts; ~90% of newborns in both groups spontaneously breathed before clamping the cord. The use of CPAP or ventilation in the first 60 s of DCC, had no effect on placental transfusion, physiological variables in the first 24 h of life, or neonatal outcomes when compared with stimulation alone.

Umbilical cord milking could offer an advantage over DCC in preterm newborns who are deemed too unstable to wait for DCC and who are at the highest risk of severe IVH and death. Compared to DCC, UCM have been associated with higher blood pressure in the first day of life (35). However, recent resuscitation guidelines (32) suggested against its routine use in preterm newborns with a gestational age lower than 29 weeks.

### The Umbilical Cord Clamping Project

A recent Italian survey (38) showed a wide variation among operators in the management of cord clamping, especially at the lowest gestational ages, where only 10% of the responders declared to perform DCC. Milking was the preferred technique to allow placental transfusion in the case of extreme prematurity, resuscitation need and cesarean delivery.

The study showed a significant correlation between the implementation of DCC and UCM and the knowledge of related benefits, the availability of obstetric-neonatal guidelines and the engagement across professions within the delivery team.

In 2016 the Italian Task Force for the Management of Umbilical Cord Clamping has been constituted to draft recommendations for the management of cord clamping in term and preterm newborns.

The aim of this document (first edition) is to provide operators involved in childbirth assistance with an updated consultation tool for optimal management of umbilical cord clamping in term and preterm newborns to standardize placental transfusion strategies in different clinical scenarios. Attention was paid to the application of DCC and UCM in extremely premature infants, according to guidelines for newborn's resuscitation (31, 32).

Preterm deliveries are classically categorized, depending on gestational age at birth, in extremely preterm (<28 weeks), very preterm (<32 weeks), moderate (32–33 + 6/7 weeks), and late preterm (34–36 + 6/7 weeks) birth.

Available studies on placental transfusion strategies, however, showed a significant overlap among these categories. For this reason, the panel decided to elaborate recommendations tailored to a unique group of patients that included extreme, very and moderate preterm newborns for whom immediate post-partum medical assistance was expected. Late preterm newborns have been considered separately because, in most cases, they do not necessitate medical interventions at birth, except for the presence of a skilled operator in neonatal resuscitation, as recommended by resuscitation guidelines in 2010 (42).

## Materials and Methods

The working group consists of obstetricians, midwifes, and neonatologists with experience in delivery room resuscitation, neonatal hematology, and cardiology, belonging to the Italian Society of Neonatology (SIN), the Italian Society of Perinatal Medicine (SIMP), and the National Federation of Midwifes (FNCO).

The Italian version of the document derives from a detailed review of the English literature until December 2017 and a summary of the recommendations already published on the subject by scientific societies and expert panels. Articles' search was performed in 2 online databases (PubMed and the Cochrane Library).

Recommendations have been drawn using the methodological approach proposed by the Grading of Recommendations, Assessment, Development and Evaluation (GRADE) Working Group (43).

After identification and prioritization of the questions to be addressed, articles were screened for further evaluation. GRADE is a consensus process that rates the quality of evidence and strength of recommendations along with values and preferences. The quality of the evidence (or confidence in the estimate of the effect) was categorized as high (where one has high confidence in the estimate of effect as reported in a synthesis of the literature), moderate (where one has moderate confidence, but there may be differences from a further elucidated truth), low (where one has low confidence in the estimate of the effect that may be substantially different from the true effect), or very low (where it is possible that the estimate of the effect is substantially different from the true effect) (44, 45).

Recommendations were based on the evaluation of the expected benefits and risks connected to placental transfusion practices and the clinical context in which they could be applied. The safety for the mother and the newborn guided the drafting of the recommendations when evidence from the literature was not conclusive. The panel discussed and approved each statement unanimously during quarterly meetings held between 2016 and 2017.

The original document has been externally reviewed and accepted by a panel of neonatologists, obstetricians, and midwifes (see Acknowledgment).

Quality of evidence and strength of recommendations are summarized in Table 1.

**Table 1:** Quality of evidence and strenght of recommendations.

**QUALITY OF EVIDENCE**	
High quality	A
Moderate quality	B
Low quality	C
Very low quality	D
**STRENGTH OF RECOMMENDATIONS**	
Strong recommendation: the desirable effects of adherence outweigh the undesirable effects	1
Weak recommendations: the desirable effects of adherence probably outweigh the undesirable effects, although the trade-offs are uncertain	2

### Delayed Cord Clamping in Vaginally Delivered Term Newborn

The meta-analysis by McDonald et al. (14) demonstrated that DCC (various definition: 1 to 5 min after birth, when cord stop pulsing, after placental descending) improves hemoglobin concentration at birth [3 studies, (RR −2.17 95% CI −4/−0.28)], iron stores at 3–6 months of age [(5 studies) RR 2.65; 95% CI 1.04–6.73], and birth weight [(12 studies) RR −101.2; 95% CI −158/−44)]. Andersson et al. (46) demonstrated that 3 min DCC improved ferritin concentration (117 vs. 81 mcg/l, *p* < 0.001) and reduced the prevalence of iron deficiency (0.6 vs. 5.7%, *p* < 0.01) at 4 months of age in a population where the prevalence of iron deficient anemia is low.

These positive results last up to the end of the first year of life in the case of infants born to mothers with low serum ferritin concentration at delivery (47, 48). Ashish et al. (48) demonstrated in a large Nepalese cohort of 540 infants that 3-min DCC reduced the prevalence of anemia and the risk of iron deficiency (RR 0.58; 95% CI 0.44–0.77) at 8 months of life. Hemoglobin concentration was significantly higher until the end of the first year of life in the DCC group compared to early clamping.

The position (relative to perineum) of the newborn during DCC did not seem to affect the volume of placental transfusion (49) but a recent trail associated the lower position of the newborn to a more efficient placental transfusion (50). Most of the available studies (14, 46, 51) compared various delayed clamping times (from 1 to 5 min or until cord pulsation) to ICC, and evaluated different hematological parameters. These researches unanimously concluded for a positive effect of DCC on iron stores and a possible contribution to improved fine motor performances and social domains at 4 years of age, especially in males (26). In term newborns not requiring immediate resuscitation, delayed clamping for at least 60 s is recommended (30–34), but the ideal time to clamp the cord to promote the most efficient placental transfusion in vaginally delivered (VD) newborns is still undetermined.

Delayed clamping has been associated with a slight increase in the need for phototherapy (2.74 vs. 4.36%; RR < 2%). This result was much debated, as it originates from the inclusion of unpublished data (McDonald, 1996). No differences between early and delayed clamping were found in mortality rate, Apgar score, admission to NICU, respiratory distress, breastfeeding at discharge and up to 6 months. The incidence of asymptomatic polycythemia was similar between DCC and ICC in the review by McDonald et al. (14) but significantly higher after DCC in the meta-analysis by Hutton et al. (15). Delayed cord clamping has no negative impact on the mother's health compared to immediate clamping (14).

Delayed cord clamping improves iron stores in the first months of life in term, vaginally delivered newborns with a high quality of evidence.

#### Recommendations

1. In vaginally delivered newborns, DCC is recommended for at least 60 s to optimize cardio-pulmonary transition at birth and to promote placental transfusion **(strong recommendation) (1A)**.
2. It is recommended to dry and stimulate non-breathing infants by rubbing the back two to three times to encourage spontaneous breathing before clamping, and to clamp the cord in cases of persistent apnea **(strong recommendation) (1B)**.
3. It is suggested to clamp the umbilical cord by 3 min after delivery to improve iron stores in the first 3–6 months of life, although the optimal clamping time has not been defined **(weak recommendation) (2B)**.
4. Clamping the umbilical cord at 3 min is not mandatory; a longer DCC is suggested (up to 5 min, until pulsations stop, etc.) if requested by parents **(weak recommendation) (2C)**.
5. During DCC, newborns may be placed on the mother's abdomen or chest or kept below the perineal plane **(weak recommendation) (2C)**.

### Delayed Cord Clamping in Cesarean-Delivered Term Newborns

A recent meta-analysis (52) showed that cesarean deliveries (CD) at term were associated with a higher amount of cord blood retained by the placenta [mean difference of 8.87 ml (95% CI 2.32–15.43)] compared to vaginal deliveries. Maternal arterial pressure, the absence of uterine contractions, the possibility of reverse flow from the newborn to the placenta was thought to influence placental transfusion performance (52–54).

Zhou et al. (52) observed that CD newborns had lower hematocrit (−2.9%, CI −4.16/−1.65, 7 studies 5,098 newborns), lower hemoglobin concentration (−0.51 g/dl, CI −0.74/−0.27; 7 studies, 6,563 newborns), and lower red blood cells count (−0.16 × 10 12, CI −0.30/−0.01; 3 studies, 3,858 newborns) compared to VD newborns. The difference in hematocrit was more pronounced in elective CD than in cesarean section performed in labor; recently, Glasser et al. (55) confirmed these results. The timing of the cord clamping was not specified in the studies included in the meta-analysis.

At present, recommendations on DCC in term newborns do not distinguish between vaginal and cesarean deliveries even though no studies explored the effects of DCC in exclusively CD newborns and only a few of them included this category of patients (56–58).

Ceriani et al. (56) recruited 266 term newborns to ICC or DCC for 1 or 3 min, respectively; about 30% of patients in each group were born by CD. The authors demonstrated that DCC augmented hematocrit in the physiologic range at 6 h of life. Patients in the 3-min DCC group showed a non-significant prevalence of NICU admission compared to other groups (8.7 vs. 5.5% in the 1-min DCC and 4.3% in the ICC group); however, these latter results should be interpreted with cautions, as there was no subgroup analysis for CD newborns. Ertekin et al. (57) demonstrated higher serum ferritin concentration at 2 months of age in 150 term newborns randomized to DCC (90–120 s) or ICC. The significantly higher rate of CD in the DCC group (30 vs. 4%, *p* < 0.001) may have affected results but confirmed the effectiveness of DCC on iron stores in CD term newborns. Recently, Andersson et al. (58) compared 64 term infants born by elective CD and recruited to 30 s DCC to a historical cohort of VD newborns whose cord was clamped by 3 min after birth (46). The authors found no differences in hematological parameters at 6 months of age between groups suggesting that, in term CD newborns, cord clamping after 30 s from birth was sufficient to ensure iron status like that achieved by VD newborns after 180 s DCC. There are no reported side effects of DCC in cesarean-delivered newborns and their mothers.

There are few data supporting DCC longer than 1 min in cesarean-delivered newborns to improve iron stores in infancy; the higher rate of NICU admission associated to 3 min DCC in the study by Ceriani et al. (56) should be further explored.

No studies compared different positions of the newborn during DCC; infants can be placed between maternal legs or beside the maternal abdomen.

Overall, DCC by 30–60 s in CD term newborns is associated with improved iron stores in the first weeks after birth, with a moderate quality of evidence.

#### Recommendations

1. It is suggested to delay cord clamping for at least 30 s and up to 60 s after birth to improve iron stores in newborns not requiring resuscitation **(weak recommendation) (2B)**.
2. It is recommended to dry and stimulate non-breathing infants by rubbing the back two to three times to encourage spontaneous breathing before clamping, and to clamp the cord in cases of persistent apnea **(strong recommendation) (1B)**.
3. In the case of DCC longer than 1 min, it is recommended to ensure the presence of a skilled operator in neonatal resuscitation to evaluate the feto-neonatal transition **(weak recommendation) (2D)**.

### Cord Milking in Vaginally and Cesarean-Delivered Term Newborns

There are only a few studies (13, 59–62) that evaluated the effect of UCM in term newborns, most of them including both vaginal and cesarean deliveries, for a total number of about 500 infants receiving UCM, mostly with cut-cord technique. Recently, a pilot trial demonstrated that milking the intact cord provided a greater blood volume compared to the cut-cord technique (63).

Cord milking was associated to improved iron stores in the first weeks of life (59, 60), compared to ICC; however, data on the effects of UCM in the first months after birth are scant (61).

Erickson-Owens et al. (59) randomized 24 CD term newborns to ICC or intact-cord UCM. Infants in the intervention group showed a higher hematocrit at 48 h of life, without differences in the Apgar score and the bilirubin peak.

Upadhyay et al. (60) enrolled two hundred newborns with gestational age ≥35 weeks, to ICC or c-UCM; more than fifty percent (56–66%) were cesarean-delivered. The prevalence of maternal anemia was around fifty percent in both groups. Milking the cord was associated to higher hemoglobin and blood pressure in the first 48 h of life and higher hemoglobin [11.9 (1.5) gr/dl vs. 10.8 (0.9) gr/dl, *p* < 0.05] and ferritin concentrations at 6 weeks of age [355.9 (182.6) mcg/l vs 177.5 (135.8) mcg/L, *p* < 0.05], compared with the control group.

The positive effects of c-UCM on iron stores lasted up to 6 months of age in a prospective study on 200 term newborns (61). The final analysis included 179 newborns; CD rate was between 52 and 59%. Mean serum ferritin was significantly higher in the intervention group (113.9 ± 43.8 ng ml^−1^ vs. 70.8 ± 39.5 ng/ml, *P* < 0.001), compared to controls. The efficacy of c-UCM did not vary with the maternal anemia status. In two other studies, DCC (60–90 s) was compared to other placental transfusion strategies (13, 62).

The comparison of different placental transfusion strategies in term newborns was inconclusive.

Jaiswal et al. (62) found no significant difference in ferritin and hemoglobin concentrations at 6 weeks of age in a cohort of term newborns (100 patients per arm, 80% born by CD) randomized to DCC (60–90 s) or c-UCM.

Yadav et al. (13) in a 3-arms randomized trial compared c-UCM to DCC and DCC followed by cut-cord UCM (100 patients per arm, gestational age ≥ 37 weeks, 40% CD). The most efficient technique to improve serum ferritin level at 6 weeks of age was the combined intervention of DCC plus c-UCM, followed by the DCC technique. The authors did not report side effects in the DCC + UCM group.

None of the previous studies provided a separate analysis by type of delivery.

Some authors suggested to milk the uncut cord if this is still full at the end of the time allotment (5) but this option has not been evaluated in clinical trials.

To our knowledge, the uncut UCM technique has not been compared to DCC in VD term newborns. The panel estimated that UCM (both techniques) in healthy vaginally-delivered term newborns should not replace a more physiological placental transfusion obtained by DCC.

Overall, c-UCM improves iron stores in CD term newborns in the first weeks of life, compared to immediate cord clamping, with a very low quality of evidence.

It is not possible to recommend the best milking technique to adopt. The uncut UCM technique may probably result in better transfusion performance but required further evaluation, both in VD and CD term newborns.

#### Recommendations

##### Cord Milking in vaginally delivered term newborns

Umbilical cord milking (both techniques) is not suggested as an equal alternative to delayed cord clamping in VD newborns not requiring resuscitation at birth **(weak recommendation) (2C)**.

##### Cord milking in cesarean-delivered term newborns

In CD newborns, if delayed cord clamping is not feasible, umbilical cord milking is suggested as a valid alternative to promote placental transfusion and improve iron stores in the first weeks of life. **(weak recommendation) (2C)**.

### Delayed Cord Clamping in Late Preterm Newborns

Delayed cord clamping in late preterm infants (34 ^0^/7–36 ^6^/7 weeks of gestational age) varied from 30 to 180 s after birth (64–72).

During DCC, infants were positioned on the mother's abdomen (64, 65), below or at the level of the perineal plane (66–72); no comparative studies evaluated the extent of placental transfusion based on different newborn's positions.

Strauss et al. (65) documented a significant increase in the circulating red blood cells volume in VD, but not CD, 30–36 weeks preterm newborns after 60 s of DCC compared to early clamping (42.1 ± 7.8 vs. 36.8 ± 6.3 ml/kg, *p* = 0.04). Salae et al. (66) randomized 86 VD late-preterm newborns to 120 s DCC or early clamping, demonstrating improved hemoglobin concentration at 48 h of life in the DCC group. Similar results were obtained by Ultee et al. (64) in a randomized trial on 37 VD late preterm newborns (18 in DCC group and 19 in the ICC group), where 180 s DCC was associated to higher hemoglobin concentration at 10 weeks of life.

Ranjit et al. (67) randomized 100 preterm newborns (30–36+6 weeks, of gestational age, mean gestational age 34 weeks, 47% CD) to ICC or 120 s DCC. The latter cohort showed higher mean hematocrit (27.3 ± 3.8 vs. 31.8 ± 3.5%, *p* < 0.00) and mean serum ferritin (136.9 ± 83.8 vs. 178.9 ± 92.8 ng/ml, *p* = 0.037) at 6 weeks of age compared to ICC. Other studies that included late preterm newborns confirmed that clamping the cord 30–60 s after birth increased blood pressure in the first 24 h after birth and hemoglobin/hematocrit values at different time points, up to 10 weeks of age if their cord were clamped after 30–60 s from birth (60, 69–72). In the trial by Ashish et al. (48) term and late preterm vaginally delivered newborns [mean GA(SD): 39.2 (1.1) weeks] were randomized to ICC or 180 s DCC. The incidence of iron deficient anemia was significantly lower in the DCC group; however, the number of late preterm infants was not provided by the authors.

Most of the studies included late preterm newborns, but only a few of them addressed to this specific category, with a very low number of patients recruited (64, 66, 72). The available data do not allow to differentiate the effect of DCC by mode of delivery. Overall, the quality of evidence was considered low or very low for the outcomes considered (iron store and hemoglobin concentration in the first weeks of life).

#### Recommendations

1. In vaginal and cesarean-delivered infants not breathing at birth, it is recommended to dry and stimulate by rubbing the back two to three times to encourage spontaneous breathing before clamping, and to clamp the cord if the baby continues not to breath **(strong recommendation) (1B)**.
2. It is recommended the presence of personnel with neonatal resuscitation skills to evaluate the newborn in the transition phase **(strong recommendation) (1B)**.

##### DCC in vaginally delivered late preterm newborns

1. It is suggested clamping the cord between 60 and 180 s in vaginally delivered newborns, not requiring resuscitation at birth, to improve hemoglobin concentration and iron stores in the first weeks after birth **(weak recommendation) (2C)**.
2. It is suggested placing newborns from vaginal delivery at the perineal level or below for the first 30 s of DCC, then on maternal abdomen **(weak recommendation)**.

##### DCC in cesarean-delivered late preterm newborns

1. It is suggested clamping the cord between 30 and 60 s in cesarean-delivered late preterm newborns not requiring resuscitation **(weak recommendation)**.

### Cord Milking in Late Preterm Newborns

Only one study that included late preterm infants evaluated the effects of UCM (73), with the cut-cord technique.

Kumar et al. (73) demonstrated in a randomized trial (32–36+6 weeks of GA, mean 34.5 weeks, 100 patients per arm, 61 and 56% CD newborns in the ICC group and DCC group, respectively) that c-UCM improved hemoglobin and ferritin concentration at 6 weeks of life, compared to early clamping. No studies compared UCM with DCC in selected populations of exclusively late preterm newborns.

No data are available on the extent of placental transfusion with milking techniques, differentiated by mode of delivery.

Umbilical cord milking improves iron stores in the first weeks after birth in late preterm infants, with a very low quality of evidence.

Delayed cord clamping and milking were not associated with adverse events both in newborns and in the mother.

#### Recommendations

In vaginally and cesarean-delivered late preterm newborns, when DCC is not feasible, UCM is suggested as a valid alternative to improve iron stores **(weak recommendation)**.

### Placental Transfusion in Newborns Less Than 34 Weeks of Gestational Age

#### Delayed Cord Clamping

Delayed cord clamping in very preterm infants is defined as a clamping that occurs between 30 and 120 s after birth; DCC longer than 120 s has not been tested.

The most recent results on the effect of DCC in preterm infants were published in 2017.

The Australian APTS trial (24) that enrolled 1,566 newborns with GA < 30 weeks failed to demonstrate any benefits deriving from delayed clamping for 60 s or more when compared to immediate cord clamping. Specifically, the authors found no difference in the incidence of severe IVH, hospital mortality, severe brain injury, late-onset sepsis, retinopathy of the prematurity, necrotizing enterocolitis, and chronic lung disease between early and delayed clamping groups.

This study did not foresee interventions to promote breathing during DCC and may explain the high protocol violation rate (about ¼ of subjects randomized to DCC received ICC) mostly due to medical concern about the infant. The exclusion of the most critical newborns may have affected the results. Indeed, statistical analysis has not been performed on the effective treatment received by the patient but based on the treatment expected by randomization, which may have introduced an attrition bias, caused by the unequal loss of patients in the DCC group.

The second significative study was the meta-analysis by Fogarty et al. (25) that included 2,834 preterm infants (996 infants ≤ 28 weeks gestation) from 18 randomized trials (including the APTS trial) of delayed vs. early cord clamping. This study provided high quality evidence that DCC (mostly ≥ 60 s) reduced hospital mortality by 30%, in newborn ≤ 28 weeks GA (RR 0.70, 95% CI −0.09 to −0.01, number needed to benefit 20), compared to ICC and reduced the proportion of infants having blood transfusion by 10% (95% CI 6 to 13%, *P* < 0.00001). Subgroup analyses showed no differences between randomized groups in Apgar scores, intubation for resuscitation, admission temperature, mechanical ventilation, intraventricular hemorrhage, brain injury, chronic lung disease, patent ductus arteriosus, necrotizing enterocolitis, late-onset sepsis, and retinopathy of prematurity.

The authors explained the positive impact of DCC in preterm newborns with the reduction of unnecessary and potentially harmful medical interventions in the first hours and days of life that could influence short and long-term outcomes through the activation of the inflammatory cascade and oxidative stress, the increased risk of infections and lengthening of hospitalization.

Many authors observed that cord clamping should be individualized and based on clinical conditions, rather than on a pre-defined ideal clamping time (7–9).

Ventilation is the key strategy of neonatal resuscitation (74) but most very and extremely preterm infants start breathing, spontaneously or after stimulation, before 1 min after birth and could safely “wait” before clamping the cord (39–41). In the case of apneic newborns, promoting spontaneous breathing through gentle stimulation during the first 30–60 s of delayed clamping may improve feto-neonatal transition.

Indeed, first steps of stabilization at birth have been associated with reduced peripartum mortality and mask ventilation (75, 76); in breathing term and late preterm newborns, every 10 s of DCC was associated with a 20% reduction in the risk of death or hospitalization (40).

**Figure 1:**
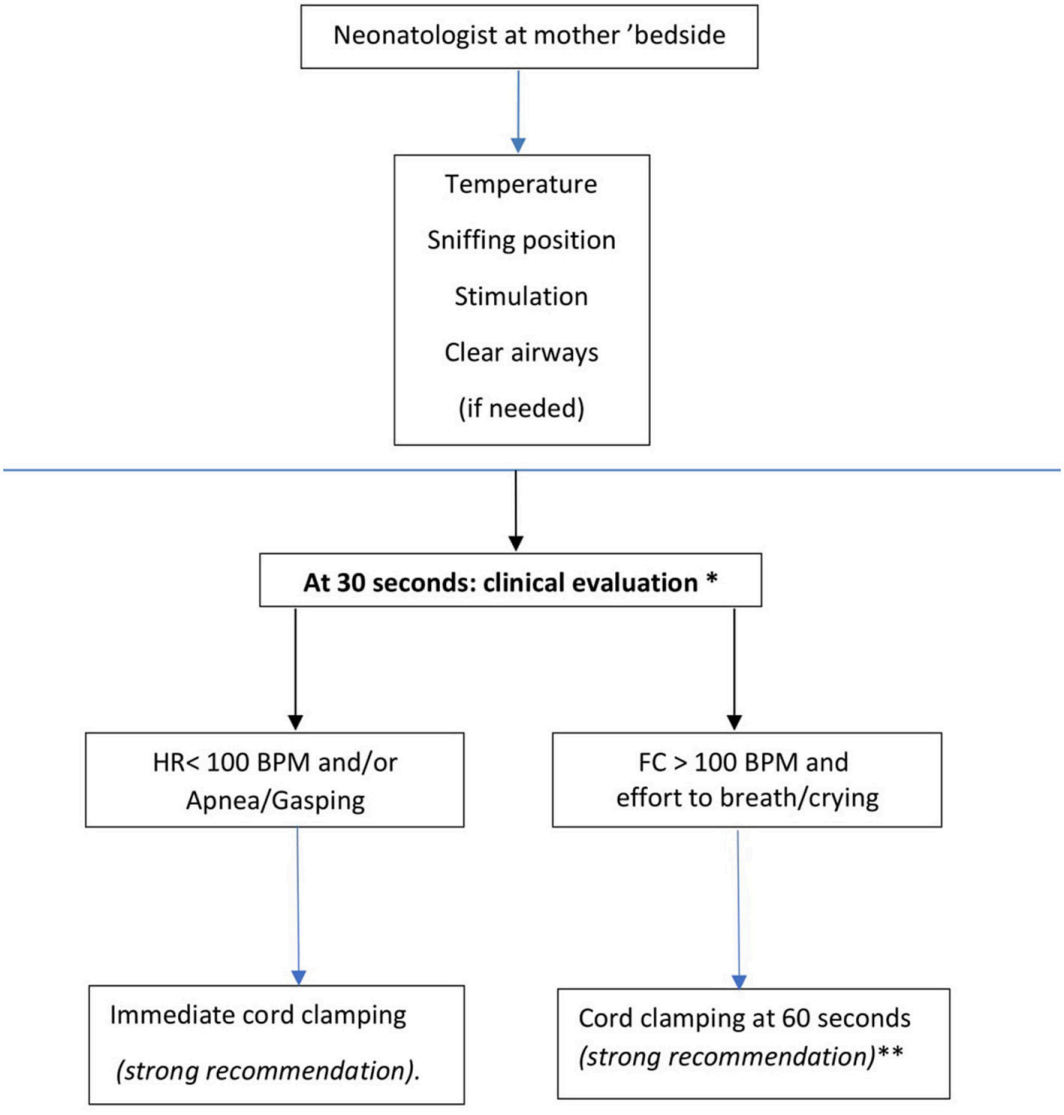
Bedside evaluation flow-chart in the case of very preterm newborns (<34 weeks). *Milking the umbilical cord is suggested when clinical evaluation bedside is not feasible, (weak recommendation). **It is suggested to clamp the cord between 90 and 120 seconds in vaginally delivered newborn with gestational age between 29 + 0 and 33 +6 weeks_(Weak recommendation).

According to these concepts, the working group proposed a flow chart (Figure 1) for premature deliveries that foresees the presence of the neonatologist at mother's bedside to safely provide at least 30 s of DCC to all newborns, while ensuring the first steps of stabilization. This model does not, therefore, create significant contradictions between neonatal resuscitation guidelines (31, 32, 42) and placental transfusion maneuvers and does not require specific equipment. A professional figure (timekeeper) responsible for checking and communicating the time elapsed from birth is suggested by various authors (5).

Delayed cord clamping increased the incidence of polycythemia in preterm newborns [risk difference 3% (95% CI: 2 to 5%)], and the incidence of jaundice (mean difference in peak bilirubin +4 μmol/l) without increasing morbidity or the need for exchange transfusion (25).

Preliminary results of long-term follow-up showed a positive correlation between DCC and improved motor function at 18–22 months of corrected age, compared to ICC (77).

Delayed cord clamping, compared to ICC, in moderate, very and extremely preterm deliveries improved outcomes with a high level of evidence.

#### Umbilical Cord Milking and Resuscitation

Milking the umbilical cord in very and extremely premature newborns was associated with a significant decreased risk of intracranial hemorrhage of all grade IVH all grade (RR 0.62; CI 0.41–0.93) and bronchopulmonary dysplasia (RR, 0.42 [95%CI: 0.21–0.83]) in newborns with gestational age < 33 weeks, compared to ICC (11) and to reduced red blood cell transfusion in newborns of < 29 weeks of gestational age (21).

When UCM was compared to DCC, results are contradictory. Rabe et al. (78) did not find differences between UCM and 30 s DCC in a small cohort of newborns < 33 weeks of gestational age. The larger randomized study by Katheria et al. (35) that enrolled 197 preterm newborns (mean gestational age 28 ± 2 weeks, mostly cesarean-delivered) demonstrated that milking the uncut cord was associated to higher hemoglobin, blood pressure, urine output in the first 12–24 h of life compared to 45–60 s DCC.

The follow-up study of the cohort by Rabe et al. (79) showed no neurodevelopmental differences between DCC and UCM groups at 2 and 3 ¼-years follow-up while infants randomized to UCM in the study by Katheria et al. (27) had higher language and cognitive scores compared with those randomized to DCC.

In moderate, very and extreme preterm newborns, especially if cesarean-delivered, cord milking improved short-term outcomes with a moderate level of evidence, and long-term outcomes with very low quality of evidence, without undesirable effects, except for a low risk of symptomatic hyperviscosity (80). For these reasons, the working group unanimously considered uncut UCM a valid option to favor placental transfusion when DCC is not feasible.

#### Recommendations (See Figure 1)

1. In vaginal and cesarean-delivered newborns with gestational age < 34 weeks, it is recommended to delay cord clamping for at least 30”. During this period, it is recommended to ensure the maintenance of body temperature, to perform tactile stimulation, to ensure airway patency and possibly their aspiration **(strong recommendation) (1A). First** steps of stabilization at the mother's bedside should be provided by the attending neonatologist **(strong recommendation) (1A)**.
2. At 30 s of life, it is recommended to evaluate tone and breathing activity by visual inspection and heart rate by stethoscope: if the newborn is bradycardic (heart rate is < 100 bpm) apnoeic or gasping, it is recommended to clamp the cord and start ventilatory assistance maneuvers according to neonatal resuscitation procedures **(strong recommendation) (1A)**.
3. At 30 s of life, if the heart rate is > 100 bpm and active breathing or efforts to breath are present, it is recommended to clamp the cord at 60 s **(strong recommendation) (1A)**.
4. In vaginally delivered newborns with gestational age between 29 + 0 and 33 + 6 weeks, that do not require ventilatory assistance, the umbilical cord may be clamped at 90–120 s of life **(weak recommendation) (2C)**.
5. The obstetrical-neonatological team should ensure sterility during bedside maneuvers, especially in the operating room **(strong recommendation) (1A)**.
6. It is suggested to place vaginally delivered newborns at or below the perineal plane to promote placental transfusion **(weak recommendation) (2C)**.
7. It is recommended to identify within the obstetric-neonatology team a professional figure responsible for checking and communicating the time elapsed from birth **(strong recommendation) (1B)**.
8. When bed-side neonatological assistance could not be implemented, milking the uncut umbilical cord three to four times before clamping the cord is suggested **(weak recommendation) (2B)**.

### Pregnancy With Feto-Maternal Red Blood Cell Alloimmunization

Rhesus disease and, generally, feto-maternal red blood cell alloimmunization, were excluded from DCC research protocols, as it was likely that delaying clamping may increase the risk for significant hyperbilirubinemia due to the higher amount of opsonized red blood cells (RBC) transfused from the placenta to the newborn that could undergo hemolysis.

There is a single retrospective study (81) that compared the effects of 30 s DCC in a group of neonates with fetal Rh-disease and fetal anemia treated with intra-uterine transfusion to a historical cohort of patients. The authors observed that neonates in the DCC group had higher hemoglobin concentration at birth (13.4 vs. 10.2 g/dl; *p* = 0.0003), lower incidence of anemia (Hb> 12 g/dl: 70.6% vs. 25%, *p* = 0.004), reduced need for exchange transfusion without increasing the rate of pathologic hyperbilirubinemia. However, the results were not conclusive due to the differences in gestational age (GA), birth weight, mode of delivery and management of jaundice between the two study periods.

#### Recommendations

1. In newborns at risk of anemia due to feto-maternal alloimmunization, it is suggested clamping the cord within 30 s from birth, after the first breaths if these occur before 30 s from birth **(weak recommendation)**.

### Delayed Cord Clamping in HIV-Positive Pregnancy

Antiretroviral therapy during pregnancy together with intrapartum and postnatal prophylaxis can prevent perinatal transmission of HIV-infection from the mother to the newborn (30, 82, 83).

WHO guidelines (30) suggest delaying cord clamping in HIV women to improve maternal and infant health and nutrition outcomes because benefits outweigh “the theoretical, and unproven, harm” of virus transmission. Newborns exposed to antiretroviral therapy during pregnancy have a higher incidence of neonatal anemia (82); DCC could be effective in increasing hemoglobin concentration in the first weeks of life and reducing the need for iron prophylaxis in infancy. To our knowledge, only one study (84) compared the effect of 2 min DCC to immediate cord clamping on 64 paired mother-newborn peers. All mothers had a negative viral load (<1,000 copies/ml) and received antiretroviral therapy during pregnancy; all newborns were born by scheduled cesarean delivery. Infants randomized to DCC had significantly higher mean hemoglobin concentration (17.36 g/dl, range 13.1–21.1 g/dl) compared to ICC group (15.1 g/ dl, range 10.9–21.2 g/dl) and this difference persisted during the first month of life, despite iron supplementation in those with anemia. At 1 year, hemoglobin concentration was similar between the two groups and all newborns showed negative HIV-PCR up to 18 months of life.

Vaginal delivery is considered appropriate for HIV-infected pregnant women who have been maintained on combined antiretroviral therapy and who have viral loads <1,000 copies/ml at or near delivery (85). Based on this recommendation, in term or near-term newborns from HIV-mother on antiretroviral therapy and viral load of 1,000 copies/ml or less, DCC could theoretically be feasible but, based on the principle of prudence, in the absence of clinical studies, it cannot be recommended. The risk of mother-to-child transmission in HIV-infected women with high viral loads can be reduced by performing cesarean delivery before the onset of labor and before rupture of membranes, in conjunction with peripartum maternal antiretroviral therapy. In these cases, DCC may theoretically increase the risk of vertical transmission. No studies have been conducted on women with high viral load nor in those that have not received antiretroviral therapies during pregnancy.

The following recommendations are intended for HIV pregnancies in high-income countries with scheduled cesarean delivery.

#### Recommendations

1. In newborns from HIV-positive mothers with adequate antiretroviral therapy during pregnancy and HIV-RNA near or at delivery ≤ 1,000 copies/ml it is suggested to delay cord clamping for at least 30 s and up to 60 s to improve iron stores, similarly to what suggested for healthy term and late preterm CD newborns **(weak recommendation) (2C)**.
2. Immediate cord clamping is suggested in all other cases **(weak recommendation) (2C)**.

### Placental Transfusion Strategies and Twin Pregnancies

Various studies (86–88) showed that the second VD newborn twin had significantly higher hemoglobin concentration at birth and in the first days of life, compared to the first one, both in monochorionic and dichorionic pregnancies.

The higher hemoglobin concentration in the second-born from monochorionic twin has been addressed to the presence of vascular anastomoses allowing either intrapartum inter-twin blood transfusion or placenta-fetal transfusion (86); in dichorionic twins the difference in hemoglobin concentration could be related to a different time of cord clamping between the first and second vaginally delivered newborn (87).

Lopriore et al. (86) retrospectively analyzed hemoglobin concentrations at birth and after 2 days of life in 300 dichorionic and 290 monochorionic preterm twin pairs. In both dichorionic and monochorionic VD twins, second-born showed a higher hemoglobin level at birth compared with their co-twin.

In twins delivered through cesarean section, no intertwin differences in hemoglobin levels were detected.

Very few studies on placental transfusion strategies included dichorionic twin pregnancies, and none included monochorionic twins.

Mc Donnel et al. (89) evaluated 46 infants (26–33 weeks of gestational age, mostly CD) randomly assigned to 30 s DCC or early clamping. Four sets of twins were included; a twin-to-twin transfusion occurred in one case of monochorionic/diamniotic pregnancy and, in another case, the first newborn showed marked anemia due to feto-maternal hemorrhage. The authors found no difference between groups in mean hematocrit in the first hours of life.

Kugelman et al. (68) randomized 65 newborns (mean gestational age 32 weeks, 65% CD) to 30–45 s DCC or ICC. The number of neonates born from multiple pregnancies (seven twin and one triplet pregnancies) was similar in the two groups. There was no significant difference in initial hematocrit between the first and the second newborn. DCC in multiple pregnancies was not associated with anemia or polycythemia (mean hematocrit: 53.2%; range vs. 54.2%; *p* < 0.74). In one twin pregnancy and the triplet one, the hemoglobin difference was <5 g/dl.

In the recent study by Katheria et al. (41), premature infants (23 + 0–31 + 6/7 weeks' gestational age) were randomized to receive positive pressure ventilation during DCC, or DCC and stimulation. The total number of newborns enrolled was 150, 125 of whom were born by CD. Multiple pregnancies were included, but there was no subgroup analysis for this category of patients; however, no adverse events were recorded.

The APTS trial (24) included two hundred-ninety newborns (about 25% of the cohort) born from twin pregnancies, including triplets and quadruplet. As for previous research studies, data on outcomes for this category of patients lack but no side effects were reported, both from vaginal or cesarean-delivered newborns.

The quality of evidence has been considered low or very low, due to the paucity of data on this subset of patients; however, the panel considered delayed cord clamping in twin pregnancies safe for mother and the child.

No studies evaluated the effect of milking in this group of patients.

#### Recommendations

1. Delayed cord clamping is not recommended in monochorionic twins because the risk of acute intertwin transfusion at birth outweighs the undetermined benefit of delayed cord clamping in this population. **(strong recommendation) (1B)**.
2. In vaginally and cesarean-delivered term or preterm twin newborns from dichorionic pregnancy, it is suggested delaying cord clamping for at least 30 s up to 60 s **(weak recommendation) (2D)**.
3. In vaginally and cesarean-delivered infants not breathing at birth, it is recommended to dry and stimulate by rubbing the back two to three times to encourage spontaneous breathing before clamping, and to clamp the cord if the baby continues not to breath **(strong recommendation) (1B)**.

### Congenital Heart Diseases (CHD)

The hemodynamic and hematological improvements associated with DCC could theoretically advantage newborns with CHD. Indeed, the increased blood volume and hematocrit may have a positive effect, especially in cyanotic CHD, through the improvement of tissue oxygenation and increased blood flow at the level of Foramen Ovale and Ductus Arteriosus.

There is a single randomized study (90) that evaluated DCC (up to 120 s) in a population of 30 newborns with severe CHD requiring corrective intervention within 1 month of life. Most of them were born by vaginal delivery; the mean clamping time was 114 s. There was no difference in perinatal events between groups. Newborns randomized to DCC significantly increased their hematocrit in the first 72 h and reduced red blood transfusion requirement both before and after surgical correction, while the incidence of thrombotic events did not increase. The authors concluded that DCC was a safe and feasible procedure for newborns with severe CHD.

Based on the prediction of hemodynamic conditions at birth (91), CHD could be categorized into three groups:

1. CHD without prediction of hemodynamic instability at birth: interatrial and interventricular septal defects, mild to moderate degree of valvular abnormalities.
2. CHD with a slight risk of hemodynamic instability at birth: obstructions to the right or left efflux with arterial duct dependence. In these conditions, the patency of the arterial duct in the first hours after birth generally allows a physiologic cardiorespiratory and hemodynamic transition at birth.
3. CHD with a high probability of hemodynamic instability at birth could compromise postnatal transition and required immediate resuscitation:
  - Transposition of great vessels with a restrictive foramen ovale
  - Hypoplastic left heart syndrome with a restrictive foramen ovale
  - Total abnormal pulmonary venous return obstructed
  - Ebstein disease with hydrops
  - Tetralogy of Fallot with absent pulmonary valve
  - Heart rhythm disorders with decompensation.

#### Recommendations

1. DCC between 1 and 2 min is suggested in the case of vaginally delivered CHD newborns **(weak recommendation) (2C)**.
2. It is suggested clamping the cord at 1 min in the case of cesarean-delivered CHD newborns **(weak recommendation) (2D)**.
3. In the case of mild or severe risk of hemodynamic instability, (group 2 and 3), it is recommended to foresee the presence of personnel with resuscitation skills to evaluate the newborn during the transition phase **(strong recommendation) (1B)**.
4. The management of cord clamping in the case of severe CHD (group 3) should be discussed prenatally by a multidisciplinary team (obstetrician, neonatologist, and cardiologist) according to the predictable resuscitation needs. **(weak recommendation) (2C)**.

### Blood Gas Analysis and Delayed Cord Clamping

Blood gas analysis from the umbilical artery is a tool to assess the metabolic status of the fetus (92). It provides quality control of obstetric care and has medicolegal implications (93). The recommended technique is to collect the blood sample from a double-clamped segment of the umbilical cord immediately after delivery of the newborn (94).

Scientific Societies (95–97) recommend performing venous and arterial cord blood gas analysis in the following situations: fever in labor; meconium-stained amniotic fluid; abnormal CTG tracing; cesarean section performed for fetal compromise; uncompensated maternal thyroid diseases; operative delivery; shoulder dystocia and twin delivery. Most of these cases, where fetal distress is suspected, and blood gas analysis recommended within a few seconds from birth, are associated with an adequate post-natal cardio-respiratory adaptation, and DCC could be safely performed. Various studies, however, demonstrated that cord acid-base status significantly worsens with time.

Wiberg et al. (98) evaluate cord blood gas analysis in 70 vaginally delivered term newborns, sampled at birth and every 45 s until cord pulsations stopped. They found a significant, time-dependent decrease of arterial pH, bicarbonates, and BE, and a significant increase of PaCO2, PO2 and lactate from T0s to T90s, with the most pronounced changes between T0s andT45s. Valero et al. obtained similar results (99).

De Paco et al. (100) found no significant differences in acid-base status evaluated in paired cord blood samples obtained immediately after birth or after to 2-min DCC in a cohort of term newborns. This methodological difference, compared to other studies, together with the small sample size, may explain the observed discrepancies.

All these studies included infants who did not require resuscitation at birth; whether the effect of DCC on arterial cord pH in non-vigorous infants would be similar is an important question requiring further investigations.

A possible solution that takes both needs into account (blood gas determination immediately after birth and delayed cord clamping in vigorous infants) is to perform blood gas analysis on the unclamped umbilical cord. Andersson et al. (93) showed that this technique is a feasible alternative method for obtaining umbilical blood samples within 30 s from birth without reducing the proportion of valid blood gas samples and without significant differences in blood gas parameters compared to the double-clamped technique.

Di Tommaso et al. (101) demonstrated that, in neonates from uncomplicated pregnancy and labor, blood gas analysis obtained from unclamped cords immediately after birth provides reliable indications on the state of fetal oxygenation without altering the accuracy of the analysis, when compared to the clamped technique, especially on the arterial side.

The quality of evidence was considered high for the outcome “reliability of cord blood gas analysis during delayed cord clamping.”

#### Recommendation

1. It is recommended performing umbilical artery gas analysis on double-clamped cord immediately after birth when neonatal asphyxia is expected, to obtain the most objective determination of the neonatal metabolic condition at of birth **(strong recommendation) (1A)**.
2. When cord gas analysis is recommended immediately after birth, but the newborn is vigorous and does not require resuscitation, umbilical artery gas analysis on the unclamped cord is a feasible alternative method **(strong recommendation) (1B)**.

### Cord Blood Banking and Delayed Cord Clamping

Umbilical Cord Blood (UCB) is currently an established source of stem cells, especially in pediatric settings, in very urgent cases and for patients with no HLA matched donor (102, 103).

In recent years, the cellularity threshold for banking has been augmented from 1.2 × 10^9^ to 1.5 × 10^9^; consequently, discarded units are increased up to over 75% (104).

Numerous factors may condition the collection of adequate UCB units; 1. weight of the newborn, 2. weight of the placenta, 3. ethnicity, and 4. collection methods (*in-utero* vs. *ex-utero*). The timing of cord clamping is the main determinant of volume and number of stem cells collected (105–107).

Early cord clamping within 30 s from delivery is associated with optimal volume and progenitor cells for UCB collection while DCC for 1 to 3 min significantly reduces cord blood volume available for collection (108, 109).

A study from the Canadian Blood Bank Service (108) showed that any cord clamping delay from birth to collection decreased the number of UCB units that met the threshold for banking; clamping the cord after 120 s correlated with the highest number of discarded cord blood units.

Differently, the National Swedish Cord Blood Bank (109) demonstrated that, although 60 s DCC was associated with a reduced volume of blood (mean difference, 8.1 ml; 95% CI, 1.3–15.0 ml), it did not impact negatively on the total number of stem cells collected (*p* = 0.1) (*in-utero* technique, threshold 1.5 × 10^9^).

Recent recommendations (110–113) stated that UCB collection must not adversely affect mother' or newborn ‘health and cord blood collection should not interfere with DCC. Since 2011, an Italian state-region agreement stated that the cord should not be clamped before 60 s, in the case of public cord blood donation.

Scientific societies (109–112) recommend providing pregnant women with unbiased information about umbilical cord blood banking options, including the benefits and limitations of public and private banks.

The following recommendations are intended for altruistic and dedicated umbilical cord blood (UCB) donations. Private cord blood banks are not allowed in Italy. Cord blood may be collected with special permission from the hospital, but units must be stored in foreign cord blood banks; transport and costs are not borne by national health service.

#### Recommendations

1. In the case of altruistic cord blood donation, it is suggested to clamp the umbilical cord after 60 s and before 120 s after birth **(weak recommendation) (2C)**.
2. In the case of directed donation for at-risk families, with the goal of maximizing the content of hematopoietic progenitors through the volume collected, it is recommended to clamp the cord immediately after birth **(strong recommendation) (1A)**.
3. Health care professionals should give written information to pregnant women and their partners of the benefits of DCC and its impact on cord blood collection and banking **(strong recommendation) (1B)**.

## Contra-indications to Delayed Cord Clamping and Umbilical Cord Milking

Although there are no maternal contraindications to DCC or UCM, there are some emergent conditions (such as massive uterine bleeding) that required immediate cord clamping to safeguard mother's health.

Fetal conditions for which DCC and UCM are contra-indicated include all cases of perinatal asphyxia when immediate resuscitation is required; other exclusion criteria include clinical situations for which concerns exist about the possible benefits deriving from a placental transfusion, and results from research studies, when present, are inconclusive.

For the following clinical situations, immediate cord clamping is recommended, although based on experts' opinions.

- Birth asphyxia secondary to hypoxic-ischemic events: placental detachment, cord prolapse, uterine rupture, shoulder dystocia, vasa previa rupture, maternal collapse, amniotic embolism, maternal cardiac arrest.
- Twin to twin transfusion (TTTS)
- Newborn from HIV-positive mother (see dedicated paragraph)
- Fetal hydrops with evidence of fetal hearth decompensation
- Doubt about the integrity of the umbilical cord
- Cesarean delivery under general anesthesia.

Theoretically, when a hypovolemic shock is suspected (i.e., shoulder dystocia, placental detachment, cord prolapse, uterine rupture) c-UCM may be an immediate source of fetal blood to be transfused while initiating resuscitations ‘maneuvers (113). Research protocols are required to evaluate this option.

### 

#### Intra-uterine Growth Restriction (IUGR)

Intrauterine growth restriction refers to a fetus with an estimated fetal weight <10th percentile on ultrasound that, because of a pathologic process, has not attained its growth potential (114). The leading cause of IUGR is uteroplacental insufficiency (115), but fetal restriction could also be associated with maternal or fetal factors. In most cases, the fetus is exposed to chronic hypoxia that leads to polycythemia due to the increased erythropoietin secretion during fetal life (116). Increased hematocrit and blood viscosity could augment the risk of neonatal morbidities, like respiratory distress, thrombosis, and cerebrovascular accidents (116).

Delayed cord clamping and cord milking have not been tested in this specific population; for this reason, the panel could not issue a recommendation on the subject. Theoretically, both techniques may worsen polycythemia (25, 81) and hyperviscosity, augmenting the risk of neonatal events, especially in infants with severe fetal restriction. The management of cord clamping in these cases should be discussed antenatally with the obstetric team.

## Summary of Recommendations

A summary of all recommendations is given in Table 2.

**Table 2:** Summary of recommendations.

Newborns ≥ 34 weeks GA	- Always dry and stimulate apnoeic infants before clamping, to encourage spontaneous breathing, and to clamp the cord in cases of persistent apnea
Vaginally delivered	- In term and late preterm newborns, delay cord clamping for at least 30 (late preterm) or 60 s (term) and up to 3 min to optimize cardiopulmonary transition and improve iron stores - Consider the mother's choice if she asks for a longer DCC - Place the newborn on the mother's abdomen/chest or kept below the perineal plane
Cesarean-delivered	- Delay cord clamping for at least 30 (late preterm) or 60 (term) s - In the case of DCC longer than 60 s ensure the presence of a skilled operator in neonatal resuscitation to evaluate the feto-neonatal transition - Milking the umbilical cord in term and late preterm newborns is a valid option when DCC is not feasible
Newborns < 34 weeks GA	- Ensure the neonatologist is at the bedside - After newborn'delivery, maintain body temperature and airway patency, stimulate, and aspirate airways when required - Evaluate breathing and cardiac frequency at 30 s: 1. Clamp the cord and resuscitate if apnoeic or bradycardic (<100 bpm). 2. If heart rate > 100 bpm and active breathing/efforts to breath: CD newborns or GA < 29 weeks: clamp the cord at 60 s. VD newborns with GA 29–33 weeks: clamp the cord at 90–120 s. - Milking the cord is a valid alternative when a newborn's evaluation at mother's bedside is not feasible - Ensure sterility during bedside maneuvers
HIV pregnancy	- Clamp the cord between 1 and 2 min in CD newborns from HIV-positive mothers with HIV-RNA ≤ 1,000 copies/mL and adequate antiretroviral therapy during pregnancy - Immediate cord clamping in other cases
Twin pregnancy	- In monochorionic twin, delayed cord clamping is not recommended - In vaginally and cesarean-delivered term or preterm twin newborns from dichorionic pregnancy, it is suggested delaying cord clamping for at least 30 s up to 60 s
Fetus with congenital heart disease (CHD)	- DCC between 1 and 2 min is suggested in VD newborns - DCC for 1 min is suggested in the case of CD - In the case of CHD at high-risk of hemodynamic instability, discuss antenatally the management of cord clamping
Cord blood banking	- In the case of altruistic cord blood donation, it is recommended clamping the cord after 60 s and before 120 s - In the case of dedicated cord blood donation, it is recommended clamping the cord immediately after birth. - Offer written information to pregnant women and their partners of the benefits of DCC and its impact on cord blood collection and banking
Blood gas analysis	- Perform umbilical artery gas analysis on double-clamped cord immediately after birth when neonatal asphyxia is expected - When cord gas analysis is recommended immediately after birth, but the newborn is vigorous and do not require resuscitation, umbilical artery gas analysis on the unclamped cord is a feasible alternative method
Contra-indications to DCC and UCM	- Birth asphyxia secondary to hypoxic-ischemic events: placental detachment, cord prolapse, uterine rupture, shoulder dystocia, vasa previa rupture, maternal collapse, amniotic embolism, maternal cardiac arrest - Twin to twin transfusion (TTTS) - HIV positive mother (see dedicated paragraph) - Rhesus disease - Fetal hydrops - Doubts about the integrity of the umbilical cord - Cesarean delivery under general anesthesia

## Author Contributions

SG conceived the project, coordinated the work of the panel between 2015 and 2016, wrote the introduction and the material and methods paragraphs, wrote and reviewed the final version of the manuscript. MD and AL wrote the first draft of paragraphs on term newborns, twins, and gas analysis. BP wrote the first draft of paragraphs on late preterms, pregnancy with feto-maternal alloimmunization, HIV-positive pregnancy and contraindications. SF contributed to writing the first draft of the paragraph on congenital heart disease. PS contributed to writing the first draft on cord blood banking. SP contributed to writing the first draft on preterm < 34 weeks and together with SG and BP conceptualize the Flow-chart. All authors approved the final version.

### Conflict of Interest Statement

The authors declare that the research was conducted in the absence of any commercial or financial relationships that could be construed as a potential conflict of interest.

## Abbreviations

BPD: Bronchopulmonary dysplasia
CD: Cesarean Delivered
CHD: Congenital hearth defect
CS: Cesarean Section
DCC: Delayed Cord Clamping
GA: Gestational Age
ICC: Immediate Cord Clamping
IVH: Intraventricular Hemorrhage
NEC: Necrotizing Enterocolitis
UCM: Umbilical Cord Milking
c- UCM: cut-cord Umbilical Cord Milking
VD: Vaginal Delivery
WHO: World Health Organization.
